# *What Quality of Care Means?* Exploring Clinical Nurses’ Perceptions on the Concept of Quality Care: A Qualitative Study

**DOI:** 10.3390/clinpract12040051

**Published:** 2022-06-30

**Authors:** Areti Stavropoulou, Michael Rovithis, Martha Kelesi, George Vasilopoulos, Evangelia Sigala, Dimitrios Papageorgiou, Maria Moudatsou, Sofia Koukouli

**Affiliations:** 1 Department of Nursing, Faculty of Health and Care Sciences, University of West Attica, Ag. Spyridonos Str., 122 43 Athens, Greece; mkel@uniwa.gr (M.K.); gvasilop@uniwa.gr (G.V.); esigala@uniwa.gr (E.S.); dpapa@uniwa.gr (D.P.); 2 Department of Nursing, Faculty of Health Sciences, Hellenic Mediterranean University, Gianni Kornarou, Estavromenos 1, 714 10 Heraklion, Greece; rovithis@hmu.gr; 3 Department of Social Work, Faculty of Health Sciences, Hellenic Mediterranean University, Gianni Kornarou, Estavromenos 1, 714 10 Heraklion, Greece; moudatsoum@hmu.gr (M.M.); koukouli@hmu.gr (S.K.)

**Keywords:** quality care, clinical nurse, nursing care, qualitative research

## Abstract

Quality is a multidimensional issue involving various features that depend on service performance and personal assessment. Clarifying the concept of quality is essential in order to further facilitate the understanding and improvement of quality in healthcare. The purpose of this study was to investigate how clinical nurses, providing care to adult medical patients, perceive and define the concept of quality nursing care. A descriptive qualitative research design was applied. A purposive sampling strategy was used to recruit nurses from the clinical sector of a general public hospital in Athens, Greece. Ten female nurses from the medical sector participated the study. Data collection was conducted through in-depth, semi-structured interviews. Conventional content analysis was used to analyze the verbatim data. Four categories were revealed from the data analysis, namely: (a) “Quality care is holistic care”, (b) “Good care is an interpersonal issue”, (c) “Leadership is crucial”, and (d) “Best care is our responsibility”. Quality care was defined as holistic care, addressing all patient needs with competency and aiming for the best patient outcomes. It was associated with communication, teamwork, good leadership, and personal commitment. By developing an in-depth and mutual understanding about what quality means, nurse leaders and practitioners may collaborate in finding common paths to support quality interventions and enhance quality nursing care in clinical practice.

## 1. Introduction

Τhe concept of quality of care has been a topic of debate among the members of the scientific community for many years, as it has been associated with various dimensions of healthcare, such as interpersonal and technical aspects of care, patient outcomes, structure, processes, and the setting of quality standards [1,2,3]. Furthermore, patient satisfaction, safety, person-centred care, staff competency, and patient involvement in decision-making are some of the indicators of a high quality of care in clinical settings [4].

Some of the most influential efforts on quality of healthcare include those of Donabedian [1], who described quality as an attribute and criterion of the healthcare provided, consisting of at least two parts: technical and interpersonal; while the Institute of Medicine [5] described quality of care as the degree to which health services for individuals and populations increase the likelihood of desired health outcomes and are consistent with current professional knowledge. Allen-Duck et al. [6] described healthcare quality as the assessment and provision of effective and safe care, which is reflected in a culture of excellence and results in the attainment of optimal or desired health. Other definitions involve concepts of effective, safe, evidence-based and person-centred care in a rapidly changing healthcare environment, in which healthcare services must be timely, equitably integrated, and efficient [4].

Despite the fact that many scientists have been working intensively to formulate a commonly accepted definition of quality care, this concept remains unclear, as quality means different things among patients, healthcare providers, and organizations [6]. Moreover, quality care appears to be a multidimensional issue involving a list of various features that depends on the level of the healthcare delivered, the health system’s goals and performance, and on stakeholders’ views and personal assessment. Accordingly, this leads to confusion about the concept of quality of care, which needs to be clarified for being able to measure and improve it [7]. In this respect, the consistent use of research concerning quality care and its related concepts seems to be imperative for the meticulous synthesis of the relevant literature.

In terms of clinical context, attributes such as effectiveness, efficiency, patient safety, best patient outcomes, and effective, continuous interaction and communication between the patient and the nursing staff, seem to be the common denominators that contribute significantly to a high quality of nursing care [8]. Ryan et al. [9], who conducted focus group sessions to identify nurses’ perceptions of quality nursing care, concluded that characteristics such as clinical competency, collaborative relationships, autonomy, supportive management, appropriate staffing, and control of nursing practice were closely related to quality of care in clinical settings. Furthermore, holistic, individualized, and family-centered care was associated with excellence in nursing practice.

As contemporary healthcare systems focus on the patient for achieving a high quality of services, factors such as personalized care, nurses’ responsiveness to patient requests, an effective patient –nurse ratio, adequate information, and accessibility were valued as important dimensions of quality care and patient satisfaction [10,11]. In addition, healthcare-quality interventions focusing upon patient outcomes and new conceptual frameworks for quality and outcomes research were developed. An illustrative example is the Quality Health Outcomes Model, which suggests that there is a reciprocal relationship among interventions, the health system, and patient characteristics that affect the desired outcomes. This model has been considered important for the quality nursing field and nursing-outcomes research, as it incorporates essential components of nursing (e.g., the ones of Fawcett’s metaparadigm: person, environment, health, and nursing) and indicators of care outcomes that are sensitive to nursing care [12].

Quality models such as the one mentioned above, have being widely used to frame research on quality of care and, as such, nursing care had to be adjusted to the novel interventions, advanced practices, and technologies raised by these challenging efforts. Accordingly, clinical nursing has been reshaped to a highly demanding sector that requires excellent nursing knowledge and the skills for achieving the best outcomes and high quality of care [13]. Within this changing environment, exploring the clinical nurses’ perceptions on what quality of care means appears to be an issue of significant interest.

A review of the literature showed that the concept of quality nursing care has not been adequately investigated and only a few studies have examined the meaning of quality care from the nurses’ viewpoint [9,14,15]. In particular, research exploring the concept of “quality care” from the nurses’ perspectives within the Greek context is lacking. Thus, the need to investigate how nurses define quality of care is imperative to fill in the knowledge gap in this area. The results of the proposed study may enrich the existing scientific knowledge on the concepts of quality and excellence in nursing care and provide opportunities for quality improvements in clinical practice.

The purpose of the study was to investigate how nurses, working in clinical settings and providing care to adult medical patients, perceive and define the concept of quality nursing care.

## 2. Methods

### 2.1. Design

A qualitative descriptive research design was applied using a conventional content analysis approach.

Qualitative research designs are particularly useful when little is known about a phenomenon and when the researchers seek to answer questions about the participants’ views, perspectives and experiences. These designs help to identify key concepts and constructs and to gain in-depth understanding of the phenomenon under study within its real social context [16]. More specifically, qualitative, descriptive design is used to provide straightforward descriptions of experiences and perceptions, in areas where little is known about a topic under investigation. It is grounded in the general principles of naturalistic inquiry, and it is considered as a valuable research method, which is distinct from other research methods. It employs a factist perspective, in that data produced from such a design convey the reality of the phenomenon or the topic under investigation [17]. In such designs, the analysis and the interpretation of the findings remain close to the data (data-near), by bringing to the fore what is really happen into the field of investigation. It is most appropriate when the researcher aims to investigate the subjective nature of the problem and the different experiences of the participants and to present the findings in a way that directly reflects the terms used in the research question [18].

As such, a, qualitative descriptive design was considered the most suitable for gaining in depth knowledge and understanding on how nurses perceive and define the concept of quality care.

### 2.2. Context and Participants

A purposive sampling strategy was used to recruit nurses from the clinical sector of a general public hospital in Athens, Greece, having a 476-bed capacity. Quality policy is supported within the hospital by the Department of Quality Control, Research, and Education. The department’s role is to highlight, promote, and monitor the implementation of the quality policies as defined by the hospital management and assist the administrators to continuously improve the quality of healthcare services by making appropriate recommendations. In this context, cooperation with nursing staff has been developed in a range of quality activities, such as monitoring of clinical errors and management of nosocomial infections. A short introductory training on quality of care is provided to the newly employed nursing personnel.

Registered nurses having at least two years of working experience in the medical sector were included in the study. This working experience was considered essential for the study participants to thoroughly discuss their views and perceptions about the quality of the care provided in their working environment.

Recruitment of study participants took place within the hospital by a member of the research team who advertised the study through informal meetings and personal contacts with the nurse managers and the staff nurses who were employed in the medical sector. At the time the survey was conducted only female nurses responded to the invitation to participate in the study. This is likely to happen not only because the number of female nurses is higher than the male ones but also because of the onset of the pandemic that limited the ability of the nurses’ participation in the study. Finally, 10 female nurses working in the medical clinics participated in the study. They all held a bachelor’s degree in nursing, while nine of them were M.Sc. graduates. Participants’ ages ranged from 30 to 47 years, and their length of employment in the clinical sector was between 2 and 22 years (Table 1). None of the participants requested to withdrew from the study.

### 2.3. Ethics

Ethical approval was gained from the scientific board of the hospital before data collection (Ref. No. 15/9-7-2019). An informed consent form was signed before conducting each interview. Relevant information on participant anonymity and confidentiality was provided. Τhe voluntary nature of the study and a participant’s right to withdraw from the study at any time without any consequences were discussed. Before each interview, permission was granted by the participants for tape recording. Personal data were protected throughout the study, and code numbers were given to participants for preserving anonymity. Interview extracts were used in order to illustrate the presentation of the findings, without including any identifying information.

### 2.4. Data Collection

Data collection was conducted through in-depth, semi-structured interviews. This kind of interviews is useful when detailed information about a person’s views is required. Semi-structed interviews allow the study participants to provide wealth of information by answering spontaneously on complex matters [19]. Data collection was carried out from February until June 2020. One member of the research team carried out all the interviews. The interviewer was appropriately trained in interviewing techniques, so close-ended and/or leading questions were avoided and participants were encouraged to provide the most detailed and rich data [20]. Four out of the ten interviews took place within the hospital in a private location, at a day and time chosen by the study participants. In March 2020, the first wave of the COVID-19 pandemic outbreak brought up a prolonged lockdown in Greece. This necessitated the application of other ways to conduct the remaining interviews. For this reason, 6 interviews were conducted via Skype. In qualitive research, the rapid development of technologies offered alternative interview modes, including e-mail, instant messaging and video calling. These modes considered to be appropriate for a variety of reasons such as cost, time, privacy and recently become a necessity due to the safety measures taken in all countries to reduce the COVID-19 pandemic. Although alternative interview modes have been discussed for both their merits (e.g., cost and time savings, safer environment) and pitfalls (e.g., time-lags on video, disconnected calls, and limited access to body language), relevant research showed that face-to-face interviews were only marginally superior to video calls, in that interviewees said more. Even so, analysis of data produced a similar number of words and a similar number of topics [21].

The interview guide included one open-ended question that invited nurse participants to describe what quality of care means for them. Additional questions were used, whenever necessary, to further explore the participants’ views concerning (a) features of good care, and (b) factors affecting the provision of quality care. Each interview lasted approximately 20 to 30 min. In total, 10 interviews were conducted. Data saturation occurred at this point of data collection, since the interviewer recognized that data were repeated in prior interviews and no new information was revealed. At this point it is important to note that emphasis was also placed by the researchers upon the concept of ‘information power’, which actually determines the sample size by the amount of information the sample provides rather than the number of the participants. ‘Information power’ is important in descriptive qualitative research, where small sample sizes are common, as a manner of ensuring that adequate information has been attained [22]. A tape recorder was used for collecting the interviews’ data. The data were transcribed verbatim and converted into written text upon the completion of each interview. All interviews were conducted in Greek. The interview conversation was translated into English, and a backward-translation technique was applied to ensure the accuracy of the translation [23].

### 2.5. Data Analysis

A conventional content analysis was used to analyze the verbatim data. In conventional content analysis, coding categories are derived directly from the data, and it is also known as inductive category development [24]. Data analysis was conducted by a member of the research team. A three-stage process was used to analyse data based on Elo and Kyngäs’ [25] process of data analysis. These stages involved preparation, organizing, and reporting. At the first stage, a process of reading the data repeatedly to achieve immersion and overall understanding of the participants’ statements was applied. Words and phrases comprised the units of analysis. At the second stage, rereading the participants’ narratives allowed the analyst to proceed to open coding using broad headings (e.g., *organizational aspects*), reflecting an explicit concept or meaning. Codes involving relative concepts were then grouped under higher-order headings (e.g., *trusting leadership*), leading to generation of categories and subcategories. At the third stage, the analysis process and the findings were reported through the formed categories and sub-categories.

For ensuring the trustworthiness of the analysis process the development of categories and subcategories was examined independently by a second analyst, a member of the authoring team, and a consensus was reached regarding the presentation of the final categories and sub-categories [24,26].

### 2.6. Credibility of Research

Member check is considered as one of the most important techniques used by the qualitative researchers to enhance a study’s credibility [27]. This was one of the methods used in the present study to ensure the credibility of the findings. Member check took place during the interview course and at the end of the conversation. In this way the participants had several opportunities to confirm that their articulations have been accurately captured. Furthermore, peer examination was applied during all steps of the present study. This allowed rigorous feedback, refinement of research methods and elimination of possible researcher bias or assumptions throughout the data analysis phase [26]. Analyst triangulation was an additional technique, which was used to enhance the credibility of the study. This involved a second analyst, who reviewed the findings of the study, in order to provide a different perspective, to uncover possible hidden concepts and, thus, strengthen the integrity of the study results. This technique also ensured that possible discrepancies in terms of coding and category formulation were resolved and a consensus was reached among the two analysts regarding the presentation of the study findings. COREQ guidelines were considered to ensure that all relevant issues for qualitative papers’ reporting were addressed [28].

## 3. Results

Ten female university-graduate nurses participated in the present study. They were all employed in the medical sector, with a minimum overall working experience of seven years. None of them had received any formal training on quality of care. The demographic characteristics of the study participants are presented in Table 1.

Study participants were asked to define what quality of nursing care means. They defined quality as a holistic approach to patient care, involving issues of communication, best patient outcome, competency, knowledge, satisfaction, and meeting the patient’s needs. The concept of quality was also related with managerial issues and personal engagement. In this respect, four categories were revealed from the data analysis, namely: (a) “Quality care is holistic care”, (b) “Good care is an interpersonal issue”, (c) “Leadership is crucial”, and (d) “Best care is our responsibility”. Nine subcategories were also formulated and were assimilated, respectively, into each appropriate category (Table 2).

### 3.1. Category A: “Quality Care Is Holistic Care”

#### 3.1.1. A1: Meeting the Patient’s Needs

*“Quality care is holistic care”:* this was the main message that the participants conveyed when asked how they define quality of care. Treating the patients as a whole and meeting their needs in a holistic way were highlighted by the participants as major issues, with regard to quality of care.

“*Quality care is holistic care. It means to be able to meet all the patients’ needs, not only to take care of his medication…*”

(p6)

They further emphasized on the significance of addressing the patient’s needs through understanding, trust, and competency, which were regarded as key features of quality and holistic care.

“*Quality care is caring for a patient as a whole with interest and understanding, meeting all his needs …*”

(p9)

“*To meet the needs of the patient, psychological or physical and to build a trusting relationship with him …*”

(p8)

#### 3.1.2. A2: Being Knowledgeable

Well-educated, skilful and knowledgeable nurse practitioners appeared to be a necessity for providing meaningful care.

“*Quality is to have the practical skills to provide meaningful care… and at the same time to have the theoretical knowledge needed…*”

(p2)

Nurses mentioned empathy and safety as integrated concepts of quality care.

“*Excellent nursing care is the theoretical and practical knowledge we get from our studies combined with empathy…*”

(p4)

“*… to apply nursing interventions based on our knowledge… to be informed and keep the patient safe…*”

(p3)

Being capable to identify and solve the patient’s problem(s) was considered as an indicator of providing a high quality of care and promoting patient recovery.

“*I like to be able to solve the patient’s problems… and when you see the patient to get better and you know that you helped in it, you know that you have done a good job*”

(p7)

#### 3.1.3. A3: Achieving Best Patient Outcomes

Contributing to a patient’s recovery and achieving best patient outcomes were mentioned as imperative features of quality nursing care.

“*… when I know that I am doing the right thing for the patient, in the right way at the right time… when I help the patient to recover… this means good care for me*”

(p4)

Nurses described these as significant aspects of personal and professional fulfillment, leading to quality care, satisfaction, and self-actualization.

“*Care is an act leading to an outcome… you care for the sick person, you try to make him feel better… and when he is cured, this is the greatest satisfaction for every nurse*”

(p5)

#### 3.1.4. A4: Being Satisfied

Nurses and patient satisfaction were referred to as essential parts of a quality-care definition. Quality care appeared to be interrelated to professional and personal satisfaction for both the nurse and the patient.

“*Quality care is when everybody is happy. Both the nurse and the patient. For doing the right thing in the right way for the patient.*”

(p1)

Furthermore, coordinated care and preserving patient privacy appeared to be important issues that determine quality of care and patient satisfaction.

“*… to have a coordinated care, to have a satisfied patient, to maintain privacy and rest, to ensure his family is next to him…*”

(p6)

### 3.2. Category B: “Good Care Is an Interpersonal Issue”

#### 3.2.1. B1: Communicating Effectively

Nurse participants were also referred to issues that constitute quality of care. Effective communication, recognition of their work from patients and other professionals, and cooperation with colleagues during work shifts, impact positively on the provision of optimal nursing care and enhanced nurses’ job satisfaction.

“*Good care is an interpersonal issue, it means having good relationships, having a nice conversation learning from people from different cultures and educational backgrounds… things that fill in your day nicely…*”

(p1)

“*To communicate with the patient and have recognition for my work, to get a thank you at the end of the day… this is quality at work*”

(p10)

#### 3.2.2. B2: Teamwork

Elimination of fragmented care through team coordination and effective teamwork was an additional asset for the provision of quality care. Meaningful communication among healthcare team members appeared to be a prerequisite for providing holistic and meaningful care.

“*… quality care should be well coordinated… to have meaningful communication with doctors… so to be able to provide holistic and meaningful care…*”

(p6)

“*(Quality is)… to have a colleague by my side, that we can work together nicely and effectively…*”

(p7)

On the contrary, lack of effective communication with other healthcare professionals, heavy workload, and inadequate time for patient care were referred to as obstructing factors that impede optimal interaction and quality care.

“*… miscommunication with other professionals, shift schedules and unclear job description… these have a negative impact on care*”

(p10)

“*I do not have enough time to deal with my patient as I should… this makes me unhappy and affects the quality of my work*”

(p4)

### 3.3. Category C: “Leadership Is Crucial”

#### 3.3.1. C1: Having a Trusting Leadership

A trusting leadership was referred to as a crucial organizational feature for introducing quality transformation at an organizational level. However, nurses stressed that this does not occur automatically but is a long-term process.

“*Leadership is crucial… They (the managers) need to support us, to recognize that we have the theoretical knowledge to provide excellent nursing care to our patients. To trust that we can change old practices to new ones. This will not happen overnight… it needs time… this may create conflicts… so it is the manager’s responsibility to decide when and how will do the change…*”

(p4)

Supportive leadership, staff recruitment, and effective resource allocation from top management are actions that may enhance quality nursing care.

“*The shortage of staff and equipment, could be resolved at a managerial level. Supportive management decisions, will really help us a lot to do our work in a better way and to provide good care…*”

(p5)

Professional development and provision of training for the nursing staff appeared to have a distinguished role in the provision of quality care.

“*The staff needs to be trained to be able to cope with critical situations to have the knowledge and the skill. This would help to deliver better quality of care*”

(p10)

#### 3.3.2. C2: Improving Working Conditions

According to the participants, working conditions have an impact on quality of care and job satisfaction. The nurses claimed that despite the fact that they love their job, poor working conditions impede the provision of quality care and cause physical and emotional exhaustion.

“*I like to be with the patient… I like nursing, I have been practicing it for so many years… but I do not like the working conditions… it all gets so difficult… and providing good care is difficult too*”

(p9)

“*… we are all tired due to staff-shortage… we are sort of resources… and because of this we are emotionally and physically exhausted. I am so disappointed with working conditions…*”

(p2)

Staff recruitment and adequate ward facilities appeared to be critical factors for improving nurses’ work and providing effective and quality care.

“*To have more nurses in each shift, to improve the nurse/patient ratio… to have more facilities in the ward, a better shift schedule… not to get so tired, this is vital for good patient care…*”

(p1)

### 3.4. Category D: “Best Care Is Our Personal Responsibility”

#### D1: Being Personally Committed

Personal engagement and a positive professional attitude were mentioned by the nurses as ways to cope with difficult situations and maintain good care. For the participants, issues of team work and strong collaboration, like an “*unbreakable chain*” among the members of the interdisciplinary team, were considered as a personal responsibility for providing good care.

“*I’m tired of it, but okay, I’ll not let it to get me down… I’m trying for the best… we all try to do our best… it’s our responsibility to provide the best care to the patient*”

(p7)

“*… it’s our personal duty to build a strong, solid cooperation between all these people (the health professionals). To be an unbreakable chain, so nothing could harm our good work, our care…*”

(p1)

Being aware of the multidimensional nature of the nursing science, consciousness of what nursing care actually is and being responsible for the care provided to the patient were reported by the participants as personal qualities that significantly impact on quality of care.

“*We need to realize what our job is about. It is a matter of character… unfortunately not everyone can understand what our job involves and how we can do things in the right way… that we should work according to protocols, to have audits, to be responsible for the care provided to the patients and for the work doing in the clinic. It is important each of us to realize that our practice, reflects to patient condition, to patients’ life, to care provided*”

(p3)

On the other hand, participants stressed that personal engagement is undermined by the difficulties they face on a daily basis in their clinical environment, as these inevitably impact the provision of quality care to a patient. Overcoming these obstacles is often done at a personal cost that negatively affects the life of nurses. The study participants suggested that organizational support for overcoming these impediments and for providing quality services is essential.

“*Of course, if the patient-nurse ratio was better, then maybe I could have some more quality time for myself within my 8 h shift, and some more quality time to devote to my patient… but this does not happen… somedays we do not have the time for a break… it is a personal cost… we provide the best nursing care at a personal cost… few things can be done if we think of the conditions within the Greek reality, but I believe that with support from the top management and with personal commitment, the care can be improved*”.

(p4)

## 4. Discussion

### 4.1. “Quality Care Is Holistic Care”: Patient Needs, Competency, Outcomes, and Satisfaction

The aim of the present study was to investigate how clinical nurses, providing care to adult medical patients, perceive and define the concept of quality nursing care.

The study findings revealed that quality of care was defined as holistic care. Nurses’ perceptions demonstrated that the holistic nature of quality care encompasses notions of skilfulness education and expertise. Furthermore, addressing the patient’s needs and building trusting relationships were associated with delivering quality nursing care. These findings confirm those of previous studies, in which quality care was described by nurses as a relationship-based care, while addressing the patient’s needs, was strongly associated to the provision of quality of care in clinical practice [9,11].

Our participants highlighted that being a competent and knowledgeable nurse practitioner appeared to be a necessary condition for providing meaningful care and achieve the best patient outcomes. In addition, our results showed that empathic nursing care, based on evidence and scientific knowledge, may lead to patient safety and satisfaction. Similar research evidence underscored that a nurse’s competence and expertise are essential to maintaining quality care and achieving optimal patient outcomes in clinical settings [29,30].

In particular, Rahmah et al. [31] demonstrated that a competent nurse may improve the quality of the care delivered and reduce incidences of missed nursing care. Chaboyer et al. [32] reported that increased patient-adverse events were associated with poor quality of care, while similar research indicated that meeting human needs through accountability, advocacy, and empathy is essential for the provision of quality nursing care in practice [14].

In addition, issues such as the significance of a well-organized care, the maintenance of patient well-being, privacy protection, and patient and staff satisfaction were underscored in our study. These features were regarded in relevant studies as critical for high quality of care, as they may reduce nurse-burnout levels and improve professional performance and clinical practice [33,34,35].

### 4.2. “Good Care Is an Interpersonal Issue”: Effective Communication and Teamwork

Interpersonal issues, such as meaningful relationships, effective communication and collaboration, work recognition from patients and colleagues, and teamwork, appear to positively impact on the provision of quality care.

Similar issues were highlighted in the study of Ryan et al. [9], in which the importance of communication and collaboration in the decisions pertaining to patient care was demonstrated. The authors further stated that ineffective collaboration among the members of the healthcare team may lead to frequently changed care plans and incomplete patient care. This was also reported by Wang et al. [36], who specified that efficient interdisciplinary communication in critical care settings is a prerequisite for delivering high-quality care, while Oshodi et al. [33] referred to teamwork as a facilitating element for providing high-quality patient care. 

Relevant research evidence reports the importance of supportive inter-professional relationships and collaborative teamwork in patient outcomes, by highlighting that recovery and discharge decisions are affected by inadequate communication among healthcare professionals [9,33]. This is in accordance with the findings of the present study, which indicated that miscommunication with other health professionals may impact the quality of patient care.

### 4.3. “Leadership Is Crucial”: Supportive Leadership and Good Working Conditions

Supportive leadership was closely associated by the participants to quality care and improvement. In the same vein, a positive working environment was valued as enhancing the provision of quality nursing care. These findings confirm those of previous studies, which demonstrate that the supportive role and the positive attitudes of ward managers were highly appreciated by nurses, as elements that facilitate nursing practice and improve quality of care in the clinical sector [33]. Asif et al. [37] demonstrated the crucial role of transformational leadership in providing optimal working conditions and empowering environments. These enable nurses to develop effective relationships with patients, leading, thus, to job satisfaction, enhancement of autonomy in nursing practice, achievement of the desired outcomes, and a high quality of care [38].

Quality of care in the present study was associated with respectable management, recognition, professional development, and staff recruitment. These findings are supported by relevant studies, stating that leaders should encourage and reward quality nursing care, while authentic leadership should create and sustain empowering work environments that reduce burnout, increase nurse job satisfaction, and improve patient-care quality [39,40].

### 4.4. “Best Care Is Our Responsibility”: Personal Commitment

Being responsible of the care provided to the patient was reported by the nurses who participated in the present study as a personal obligation that significantly impacts the quality of care. Quality care was associated with nurses’ personal engagement and positive professional attitudes. Personal qualities such as awareness, commitment, responsibility, and consciousness appeared to impact on the quality of care. The relevant literature reported that nurses with high levels of professional commitment strive to promote integral care, and well-committed nurses are needed for providing safe and effective healthcare [41,42]. Accordingly, professional commitment seems to be closely related to increased quality of care, patient safety, personal satisfaction, professional authority and the ability to make appropriate decisions [42,43]. It does not, however, depend solely on personality traits but is rather influenced by persuasive leadership styles that foster engagement [38].

Additionally, the study participants mentioned the difficulties encountered in their clinical environment, which negatively affect their personal lives and patient care. They further referred to the crucial role of personal commitment and organizational support in overcoming these problems. Previous research evidence indicates that good organizational resources and effective management are important predictors of nurses’ professional commitment and overall staff empowerment [41,44]. According to Burhans and Alligood [14], responsibility, caring, intentionality, empathy, respect, and advocacy were validated as the essence of quality nursing care. These properties can be used to inform and evaluate clinical practice and quality of care and, as such, nurse managers may develop strategies to support nurses in this direction.

The message conveyed through our study and the formed definition of “*quality of care is holistic care*” is that quality is shaped not only through typical evaluation, which is often conducted in an impersonal way, but through human understanding, personal insight, and empathy.

It is important to note at this point that the nurses in the present study, although they defined quality care as holistic care, failed to openly demonstrate an in-depth understanding of the notion of holistic care as described in the relevant literature [45]. The emotional, social, and spiritual dimensions of holistic care, were partially and not openly revealed in the nurses’ quotations. These were rather found as hidden concepts through nurses’ referrals to patient needs, well-being, understanding, and empathy. In contrast, nurses’ perceptions focused more clearly on the physical aspects of care by drawing a connection between holistic care, therapeutic outcomes, physical issues of care, and their own personal skills and personality elements. This is a trait of the dominant biomedical model used in helathcare, which gives attention to the physical dimension of care and restricts the patient’s participation in the management of disease and decision-making.

In our case, although a lack of nurses’ conscious knowledge on the notion of holistic care was indicated, the participants appeared to intuitively communicate these concepts in patient care. By referring to issues of empathy, self-responsibility, self-awareness, and understanding the patient’s needs, we realize that a humanistic approach to care is demonstrated and that the need to further educate nurses on holistic care and quality seems imperative, for consciously implementing quality and holistic care in clinical practice.

Furthermore, defining *quality care as holistic care* may reinforce the already formed perceptions that knowledge, ability, interdisciplinary communication, and personal commitment are important dimensions of the definition of quality of care. In particular, the nurses’ perceptions of what quality of care means seem to move beyond the Donabedian’s triad of structure, process, and outcomes, by valuing personal engagement and professional commitment as major issues that constitute the concept of quality of care. The definition of quality and its relevant components, as evolved through the nurses’ eyes, highlights the inextricable link between quality and the essential dimensions of nursing, as described by Fawcett [46]. It further promotes the development of models of quality healthcare, by signifying the importance of empathy and personal attachment to quality, as these appear to directly impact on the improvement of care in the clinical sector.

## 5. Limitations

The study sample consists solely of female nurses working in the clinical sector. A sample involving male nurses too, might provide additional views on the concept of quality of care. In addition, the study participants were all employed in the medical sector of one public hospital, located in Athens. For this reason, transferability of the results is limited, and applicability of the study evidence in other contexts and situations may be restricted. A future study with a sample from different clinical areas, in both public and private healthcare sectors, and from broader geographical areas would enhance the scientific knowledge in the field under investigation. Finally, during the data-collection phase, the first wave of the COVID-19 pandemic was occurring, which resulted in a prolonged lockdown in Greece. Major and immediate changes were introduced in healthcare systems globally that severely affected nursing practice. To this end, the results of the present study should be viewed, while taking into consideration these decisive changes.

## 6. Implications for Clinical Practice

Quality of care is a complex and multidimensional concept. Few studies have focused on clarifying this concept. The findings of the present study have significant implications for both clinical practitioners and nurse leaders. The knowledge evolved from this study can be used by clinical nurses to further evaluate and improve their practice. The study findings provide significant information for reframing nursing education and care, enhancing patient safety, and achieving best patient outcomes. Sharing a common understanding of what quality of care means can assist nurse leaders to develop supportive strategies for promoting nurses’ professional development, satisfaction, and empowerment. Furthermore, at an organizational level, the evidence raised from this study may contribute to creating positive working environments and supporting teamwork and commitment. Shared actions based on quality expertise can further inform clinical practice, nursing education, and competence.

## 7. Conclusions

The concept of quality of care was defined by nurses as holistic patient care, which encompasses the issues of meeting the patient’s needs, nurses’ competency and empathy, best patient outcomes, and satisfaction. Additional features, such as effective communication among healthcare professionals and patients, teamwork, and good working conditions, are inextricably linked to the concept of quality of care and the quality level of provided health services. Personal and professional commitment and supportive leadership were considered to be factors that facilitate the provision of quality care in the clinical setting. Future research will contribute to gaining further understanding and knowledge on the concept of quality and its related factors.

## Figures and Tables

**Table 1:** Demographic characteristics of study participants.

Participant Code Number	Age	Years of Working Experience	Years of Working Experience in the Medical Sector	Education
p1	47	24	9	B.Sc., M.Sc.
p2	38	12	10	B.Sc., M.Sc.
p3	47	25	22	B.Sc., M.Sc.
p4	35	15	10	B.Sc., M.Sc.
p5	31	8	2	B.Sc., M.Sc.
p6	47	25	22	B.Sc., M.Sc.
p7	37	13	9	B.Sc., M.Sc.
p8	39	14	14	B.Sc., M.Sc.
p9	42	17	14	B.Sc., M.Sc.
p10	30	7	2	B.Sc.

**Table 2:** Categories and sub-categories.

Categories	Sub-Categories
***A. “Quality care is holistic care”***	A1. Meeting the patient’s needs
	A2. Being knowledgeable
	A3. Achieving the best patient outcomes
	A4. Being satisfied
***B. “Good care is an interpersonal issue”***	B1. Communicating effectively
	B2. Teamwork
***C. “Leadership is crucial”***	C1. Having a trusting leadership
	C2. Improving working conditions
***D. “Best care is our responsibility”***	D1. Being personally committed

## Data Availability

Data generated during the present study are not possible to be shared due to issues of subjects’ privacy and confidentiality.
